# Short-term effects of real-time feedback gait training using inertial measurement units on gait parameters in older inpatients: a within-subject pilot study

**DOI:** 10.1186/s12877-026-08086-2

**Published:** 2026-08-05

**Authors:** Daichi Shimose, Takasuke Miyazaki, Hisanori Matsuura, Sota Araki, Masayuki Kawada, Ryoji Kiyama

**Affiliations:** 1 Course of Health Sciences, Graduate School of Health Sciences, Kagoshima University, Kagoshima, Japan; 2 Department of Rehabilitation, Tarumizu Municipal Medical Center, Tarumizu Central Hospital, Kagoshima, Japan; 3 Department of Rehabilitation, Faculty of Health Science, Kumamoto Health Science University, Kumamoto, Japan; 4 Department of Physical Therapy, School of Health Sciences, Faculty of Medicine, Kagoshima University, Kagoshima, Japan

**Keywords:** Biofeedback, Gait speed, Gait analysis, Gait kinematics, Wearable sensor, Statistical parametric mapping, Rehabilitation

## Abstract

**Background:**

Feedback-based gait training has been shown to improve gait performance. Feedback gait training using inertial measurement units (IMUs) has been implemented in clinical settings but is limited to examining immediate effects; long-term effects on gait parameters such as gait speed remain unclear. This pilot study aimed to investigate the short-term effects of real-time feedback gait training using IMUs on gait parameters in older inpatients.

**Methods:**

Seven older inpatients participated in this within-subject study with baseline, control, and intervention periods. During the intervention period (five times per week over one week), participants received real-time auditory feedback using IMUs to increase ankle plantar-flexion angle or leg-extension angle, defined as the ankle position relative to the hip in the sagittal plane during the late stance. The tilt angles and accelerations of the pelvis and lower-limb segments were measured using seven IMUs at each time-point. A one-factor repeated-measures analysis of variance, Friedman test, and post hoc tests were conducted to examine the short-term effects of gait training. One-dimensional statistical parametric mapping (SPM1d) was used to assess phase-specific changes across the gait cycle.

**Results:**

Gait speed and stride length increased during the feedback period with large effect sizes (*p* = 0.047; *r* = 0.91). Ankle plantar-flexion angle on the feedback side differed across baseline, pre-training, and post-training assessments (F = 4.46, *p* = 0.036; η² = 0.43), but post hoc tests showed no differences between time-points (*p* ≥ 0.065). The leg-extension angle did not differ between time-points on the feedback side (F = 1.40, *p* = 0.283; η² = 0.19). SPM1d analysis of gait kinematics, including ankle plantar-flexion and leg-extension angles, identified significant clusters during the late stance phase.

**Conclusions:**

Feedback gait training using IMUs demonstrated potential for increasing gait speed, with substantial effect sizes and phase-specific kinematic changes identified using SPM1d. These preliminary findings suggest that feedback-based gait training could modify both spatiotemporal and kinematic parameters, and provide initial evidence regarding the feasibility and potential for improving gait ability in older inpatients in clinical settings.

**Trial registration:**

University Hospital Medical Information Network Clinical Trial Registry (UMIN-CTR 000051328).

## Background

Walking ability is fundamental to activities of daily living, and its decline leads to increased fall risk and reduced social participation among older adults [1, 2]. Gait speed is a key indicator of walking ability, and gait training is widely implemented in clinical settings to improve mobility and functional outcomes. In recent years, the use of biofeedback in gait training has attracted considerable attention [3]. Biofeedback approaches that provide real-time information regarding movement during task execution have been proposed as effective strategies for facilitating motor learning and improving gait performance [4]. By presenting kinematic or kinetic information during walking, biofeedback enables participants to immediately adjust their movements, potentially enhancing the effectiveness of gait training. Such approaches may be particularly beneficial for older adults, who often exhibit decreased gait speed and impaired gait patterns associated with reduced mobility and an increased risk of falls.

Previous studies have demonstrated that providing feedback on gait parameters, such as joint angles and ground reaction forces, can improve gait patterns in healthy young adults, older adults, and individuals with post-stroke hemiplegia [5–7]. Despite these advantages, many existing biofeedback systems rely on laboratory-based motion analysis equipment, such as optical motion capture systems and force plates, which require specialized facilities and technical expertise. Walking on a treadmill differs from overground walking in terms of oxygen consumption [8] and spatiotemporal parameters [9]. Furthermore, the application of biofeedback to overground walking [10], which closely resembles daily life activities, has not been sufficiently investigated. Therefore, to facilitate the clinical application of feedback-based gait training, it is essential to develop and validate methods that enable feedback during unrestricted overground walking.

Recently, inertial measurement units (IMUs) have emerged as promising tools for gait analysis and biofeedback. IMU-based systems are portable, relatively inexpensive, and capable of capturing movement data in real-world environments, making them suitable for clinical and community-based rehabilitation [11–13]. IMUs are well suited for developing practical biofeedback interventions for older adults. A review of prior research reported that sensor-based interventions showed limited effectiveness in improving gait speed in older adults [14]. However, feedback interventions using wearable sensors in this review included balance exercises, which may have limited their effects on walking speed. This highlights the need for methods that provide direct feedback during walking. Previous studies have attempted overground gait training with feedback on late-stance phase joint angles, including ankle plantar-flexion and lower limb-extension angles, using IMUs in healthy young participants [15], patients with stroke [16, 17] and older inpatients [18]. These studies demonstrate immediate effects on joint angles, step length, and walking speed in older inpatients [18]; these studies demonstrated an immediate increase in walking speed accompanied by changes in gait kinematics and spatiotemporal parameters. However, gait training using IMUs in clinical settings has primarily focused on immediate effects [16–18], and it is necessary to investigate the long-term effects on gait parameters such as gait speed. Therefore, by confirming the short-term effects of IMU-based biofeedback gait training in clinical settings, this study may contribute to the clinical application of effective gait training aimed at maintaining gait speed in older adults while providing a foundation for future investigations of its long-term effects. This study aimed to investigate the short-term effects of gait training using an IMU-based biofeedback system in improving gait parameters in older inpatients.

## Methods

### Study design

**Fig. 1:**
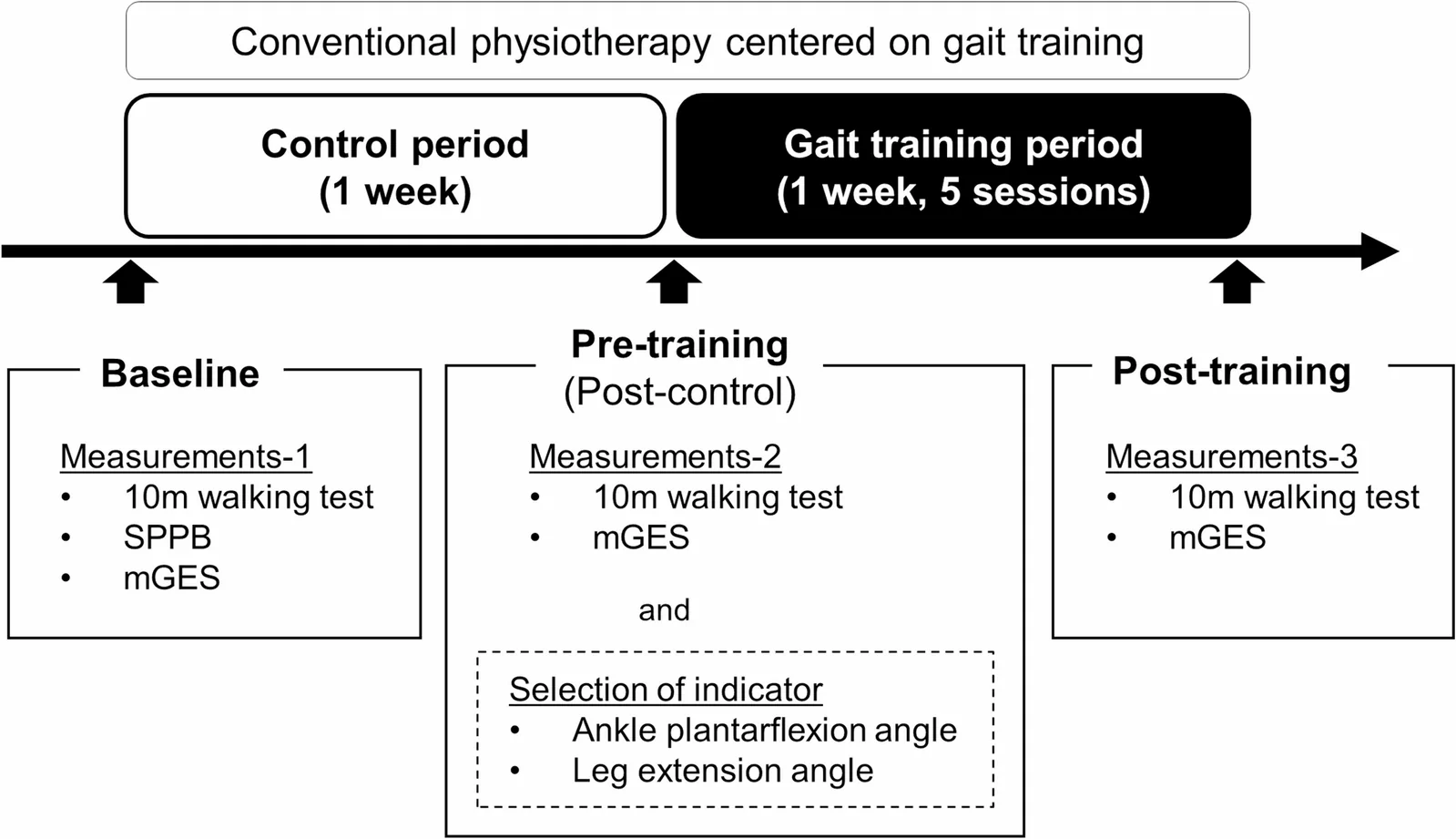
Overview of the experimental protocol

This pilot study employed a quasi-experimental within-subject design with repeated measures to investigate the preliminary effects of a short-term gait training program on gait parameters in older inpatients. The study consisted of a 1-week baseline period followed by a 1-week intervention period (Fig. 1). Gait assessments were conducted at the end of the baseline period and immediately after completion of the intervention period.

### Participants

Older inpatients were recruited from Tarumizu Central Hospital between May 2023 and November 2024. Participants were eligible if they could walk without assistive devices. The exclusion criteria were as follows: (1) severe cognitive impairment preventing the understanding of instructions; (2) neurological or musculoskeletal disorders that markedly affect gait; and (3) medical conditions contraindicating participation in gait training.

Seven participants were included in the analysis; as this was a pilot study, a formal sample size calculation was not performed. All participants provided written informed consent before participation. The study was conducted in accordance with the Declaration of Helsinki, and approved by the ethics committee of Tarumizu Central Hospital (approval no. 20 − 8).

### Study protocol

The study protocol consisted of a 1-week control period followed by a 1-week gait training period (Fig. 1). During the control period, participants received conventional physiotherapy as part of standard inpatient rehabilitation without additional structured gait training specific to the study. During the gait training period, participants underwent a structured gait training program (five times per week) in addition to conventional physiotherapy. Outcome measurements, including gait and physical function, were conducted at three time-points: baseline, pre-training (post-control), and post-training, to evaluate the short-term effects of gait training (Fig. 1).

### Feedback gait training

**Fig. 2:**
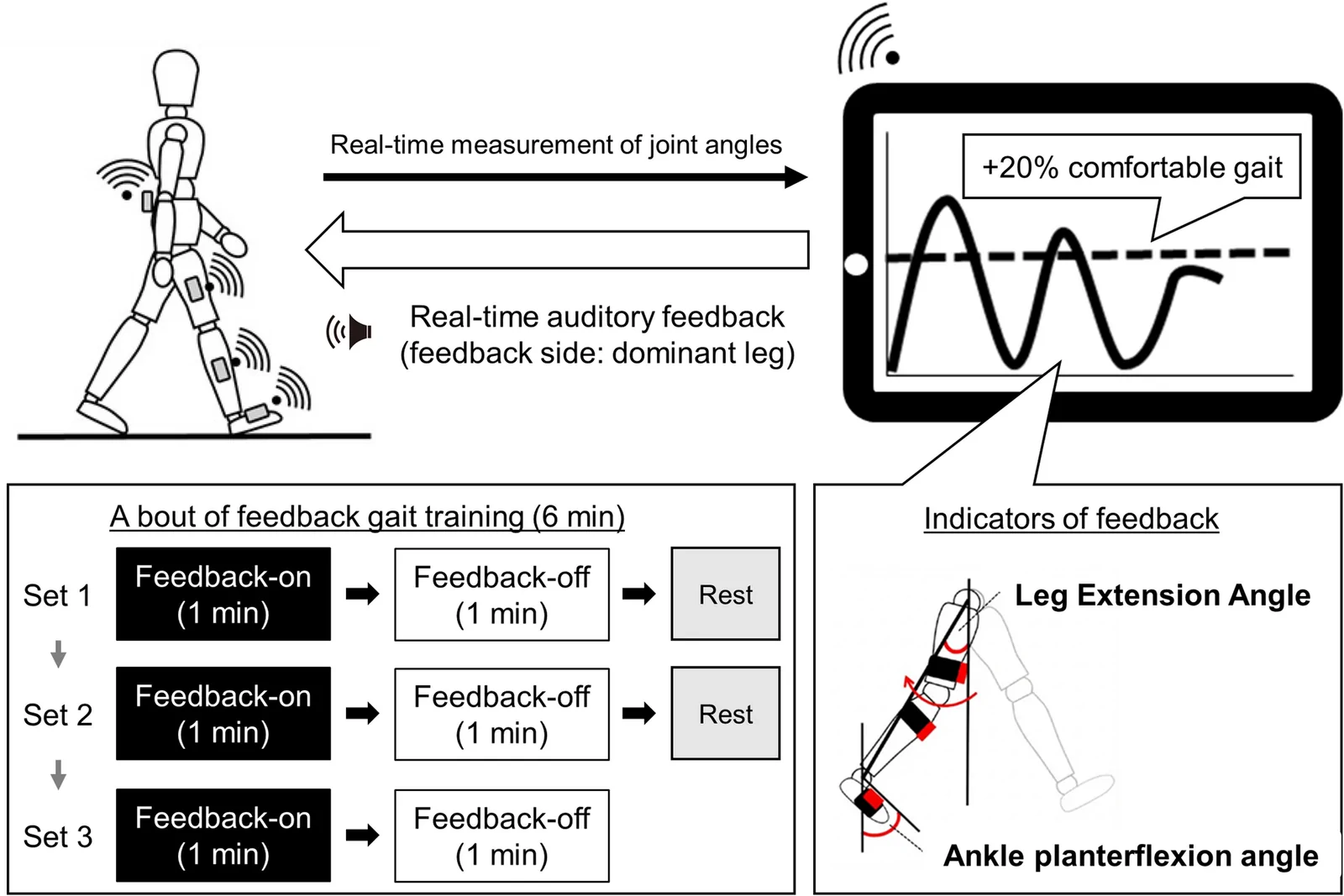
Methods of the feedback gait training using inertial measurement units

The feedback gait training system using IMUs is presented in Fig. 2. This training aimed to increase gait speed by modifying gait kinematics using real-time auditory feedback. Participants walked on a 30 m straight walkway during gait training.

Before gait training, IMUs (Movella MTw Awinda; XSens, Enschede, The Netherlands), consisting of a 3D gyroscope, 3D accelerometer, and 3D magnetometer, were attached to seven locations: the posterior sacrum, anterior aspects of both thighs and shanks, and dorsal feet [15]. The sampling frequency was set at 100 Hz. The 3-axis acceleration and tilt angles in a global coordinate system were obtained from the magnetic and inertial data using a Kalman filter in MT Manager software (4.7.2; XSens, Enschede, The Netherlands). To examine changes during gait training, gait was measured using IMUs at each time-point (Fig. 1).

IMU-based real-time feedback gait training was performed using a mobile personal computer (PC) (One-Mix3Pro, Tech-OneCo. Ltd., Tokyo, Japan), and the joint angles calculated by the IMUs were displayed on the PC (Fig. 2) [16, 18]. The PC screen displayed the joint angles calculated by the IMUs in real time, and the feedback threshold was set at a 20% increase in the peak values of each joint angle during comfortable gait before each gait training session. Participants were provided with continuous real-time auditory feedback, and a beep sound was emitted when the current joint angle reached the threshold during each gait training session (Fig. 2).

Each gait training session lasted 6 min, with auditory feedback provided intermittently between 1 min of feedback on and 1 min of feedback off. During the gait training, participants modified their gait in response to a beep sound emitted by the computer when their joint angle reached the threshold. The feedback threshold was set at a 20% increase in the peak value of comfortable gait prior to each gait training.

Two types of feedback indicators were used [16, 18]: leg extension angle, ankle position relative to the hip in the sagittal plane, and ankle plantar-flexion angle during the late stance, both of which are related to forward propulsion during walking (Fig. 2) [19, 20]. The feedback indicator was determined the day before the gait training period (Fig. 1). In detail, participants performed preliminary feedback gait training using both leg extension and ankle plantar-flexion angles for one1 min each. The angle at which the participants felt comfortable walking during the preliminary gait training was then selected as an individual feedback indicator for the gait training period.

Training sessions were conducted five times per week for 1 week, with each session consisting of three sets that alternated between 1 min of feedback on and 1 min of feedback off (Fig. 2) [16]. During the feedback-off phase, the participants were instructed to maintain the same walking pattern as during the feedback-on phase. The rest duration between sets was adjusted according to each participant’s motor function and physical condition [6].

### Outcome measures

The primary outcome measure was gait speed. Secondary outcomes included gait kinematic parameters derived from the IMU data, such as joint angles. The primary outcome was selected based on its clinical relevance to mobility and functional independence in older adults.

At each measurement-point, outcomes including walking, physical function, and self-efficacy related to walking were assessed (Fig. 1). For gait measurement, participants completed a 10 m walking test using IMUs on a 14-m straight walkway that included acceleration and deceleration areas. Measurements were conducted twice at a comfortable walking speed. Physical function was assessed using the Short Physical Performance Battery (SPPB). The SPPB is a comprehensive physical performance assessment consisting of gait speed, the five-time sit-to-stand test (FTSST), and a balance test [21]. For the FTSST, all participants used the same chair with a height of 42 cm [22]. Self-efficacy related to walking was measured using the modified Gait Efficacy Scale (mGES) [23]. As the mGES is specifically focused on walking situations in daily life, it can be widely applied in clinical and research settings to evaluate the effects of interventions aimed at improving walking ability [23, 24]. The 10-m walking test and mGES were performed at all measurement-points, whereas the SPPB was performed only at the baseline measurement (Fig. 1).

### Data analysis

The sampling frequency was set at 100 Hz. Low-pass filtering was performed on the joint angle and acceleration data were measured using IMUs, with cutoff frequencies of 10 and 20 Hz [18]. For the spatiotemporal parameters, cadence was calculated by identifying heel contact during the maximum posterior tilt angle of the sensor on the shank [25]. Stride length and gait speed were calculated based on walking time measured by the IMUs. The ankle joint angle was calculated as relative Euler angles measured from IMUs fixed on the shank and foot segments [15, 18]. In addition, the leg extension angle was determined based on the position of the ankle joint relative to the hip joint in the sagittal plane, estimated from the tilt angle matrix measured by the IMUs and the vectors of the thigh and shank segments scaled by segment length [26]. Gait data were time-normalized to 100% of the gait cycle based on initial contact of both sides. Data from the middle five gait cycles of each walking trial were analyzed. These analyses included the entire gait cycle and maximum joint angles. Data were processed using MATLAB R2020a (MathWorks, Inc., Natick, MA, United States).

### Statistical analysis

Because this was a pilot study with a small sample size, the statistical analyses were considered exploratory. Descriptive statistics were presented as means and standard deviations.

The normality of the data distribution was assessed using the Shapiro–Wilk test. Parametric statistical tests were applied to normally distributed variables, whereas non-parametric tests were used when the assumption of normality was not satisfied. Changes in gait parameters across the three time-points (baseline, pre-intervention, and post-intervention) were analyzed using repeated-measures analysis of variance (ANOVA) or the Friedman test. When significant main effects were detected, post hoc pairwise comparisons were performed using the Bonferroni correction. Effect sizes were calculated using partial eta squared (η²) for ANOVA, Kendall’s W for the Friedman test, and Cohen’s d and r for pairwise comparisons. Effect sizes were interpreted according to Kendall’s W (small, 0.10; medium, 0.30; large, ≥ 0.50), and Cohen’s d (small, 0.20; medium, 0.50; large, ≥ 0.80) and r (small, 0.10; medium, 0.30; large, ≥ 0.50) [27]. Correlation analysis was conducted between the maximum values and changes in gait parameters during each period and physical function. Additionally, to compare longitudinal changes in joint angles throughout the gait cycle before and after the intervention, one-dimensional statistical parametric mapping (SPM1d) t-tests were performed using Python 3.12.4 (Python Software Foundation, Wilmington, DE, USA) and its open-source code (http://www.spm1d.org/, accessed November 28, 2024). This method enables a detailed evaluation of joint angle changes throughout the gait cycle [28, 29]. Statistical tests were conducted using Python 3.12.4, with a significance level of 5%.

## Results

### Participant characteristics and gait training parameters

A total of seven older inpatients (mean age, 77.57 ± 9.45 years) participated in this study. The baseline characteristics are summarized in Table 1. Primary diagnoses included musculoskeletal disorders (*n* = 3), internal medical conditions (*n* = 3), and cerebrovascular disease (*n* = 1). The mean baseline walking speed was 0.87 ± 0.14 m/s, and the mean SPPB score was 8.43 ± 2.19 points. During gait training, five participants received ankle-focused biofeedback, whereas two participants received hip-focused biofeedback. All participants completed the protocol without adverse events.

**Table 1:** Baseline characteristics of participants

Case	Age	Sex	Disease	SPPB (points)	FTSST (s)	Gait speed (m/s)	Feedback Indicator	Feedback Side
1	89	M	Locomotive syndrome	10	9.37	0.97	LEA	Left
2	89	F	Femoral fracture (post-operative)	6	Unable	0.99	LEA	Right
3	70	M	Lumbar spinal stenosis (post-operative)	11	12.22	0.70	ANK	Right
4	72	F	Myocardial infarction	9	15.02	0.91	ANK	Right
5	64	M	Subarachnoid hemorrhage	11	12.00	0.97	ANK	Right
6	73	M	Diabetes Mellitus	6	Unable	0.94	ANK	Right
7	86	F	Lumbar vertebral fracture	6	Unable	0.60	ANK	Right
Mean ± SD	77.57 ± 9.45			8.43 ± 2.19	12.15 ± 1.99	0.87 ± 0.14		

Values are expressed as mean ± SD

*SPPB *Short physical performance battery, *FTSST* Five-time sit-to-stand test, *M* Male, *F* Female, *LEA* Leg extension angle, *ANK* Ankle planterflexion angle

### Changes in spatiotemporal gait parameters

Comparisons of longitudinal changes in spatiotemporal gait parameters across the three time-points (baseline, pre-training, and post-training) are summarized in Table 2.

**Table 2:** Comparisons of gait parameters and self-efficacy across time

	Control period	Gait training period		Effect size
	Baseline	Pre-training	Post-training	
Spatio-temporal gait parameters				
Walking speed (m/s)	0.94 (0.80, 0.97)	0.94 (0.86, 1.06)	1.08 *† (1.00, 1.26)	W = 0.88
Stride length (m)	0.99 (0.84, 1.02)	1.00 (0.87, 1.04)	1.07 *† (1.01, 1.24)	W = 0.80
Cadence (steps/min)	115.08 (108.17, 118.22)	117.32 (112.33, 120.83)	118.57 (116.83, 122.48)	partial η²=0.51
Kinematic Parameters in late stance				
Ankle plantarflexion angle (°)				
Feedback side	17.71 (14.99, 24.60)	23.14 (19.05, 33.41)	22.43 (16.65, 32.68)	partial η²=0.43
Non-feedback side	19.97 (16.77, 23.82)	23.17 (17.09, 29.96)	31.03 **†** (22.75, 33.05)	partial η²=0.49
Leg extension angle (°)				
Feedback side	17.33 (15.96, 18.05)	17.04 (15.87, 19.98)	20.38 (16.00, 20.48)	partial η²=0.19
Non-feedback side	17.46 (15.26, 19.67)	17.91 (14.34, 19.41)	18.43 (14.59, 26.01)	W = 0.25
Self-efficacy				
mGES	70.00 (49.50, 84.00)	72.00 (48.50, 94.00)	74.00 (61.50, 92.50)	partial η²=0.23

Values are presented as median (first quartile, third quartile)

Effect sizes are reported as partial η² for ANOVA and Kendall’s W for Friedman tests

mGES, modified Gait Efficacy Scale; Control period, comparisons between values measured at baseline measurement and those obtained pre-training measurement; Gait training period, comparisons between values measured at pre-training measurement and those obtained post-training measurement

* Significant difference between pre and post periods (gait training period) by post-hoc test

† Significant difference between baseline and post-training period by post-hoc test

Gait speed differed significantly across baseline, pre-training, and post-training assessments (χ² = 12.29, *p* = 0.002; W = 0.88). Bonferroni-corrected post hoc tests indicated that walking speed increased significantly from baseline to post-training (*p* = 0.047; *r* = 0.91) and from pre- to post-training (*p* = 0.047; *r* = 0.91), whereas no significant difference was observed between baseline and pre-training (*p* = 0.094).

Stride length also differed significantly across baseline, pre-training, and post-training assessments (χ² = 11.14, *p* = 0.004; W = 0.80). Bonferroni-corrected post hoc tests indicated that stride length increased from baseline to post-training (*p* = 0.047; *r* = 0.91) and from pre-to post-training (*p* = 0.047; *r* = 0.91), whereas no significant difference was observed between baseline and pre-training (*p* = 0.328).

Cadence also differed significantly across baseline, pre-training, and post-training assessments (F = 6.16, *p* = 0.014; η² = 0.51). Bonferroni-corrected post hoc tests indicated that cadence did not change between time-points (*p* ≤ 0.056).

### Changes in gait kinematic parameters

Comparisons of longitudinal changes in gait kinematics between time-points are summarized in Table 2.

The ankle plantar-flexion angle on the feedback side differed significantly across baseline, pre-training, and post-training assessments (F = 4.46, *p* = 0.036; η² = 0.43). Bonferroni-corrected post hoc comparisons did not show statistically significant differences between time-points (*p* ≥ 0.065). However, effect size analysis revealed a small effect during the control period (baseline to pre-training; d = 0.44) and a moderate effect during the intervention period (pre-training to post-training; d = 0.74). The ankle plantar-flexion angle on the non-feedback side also differed significantly between time-points (F = 5.64, *p* = 0.019; η² = 0.48). Bonferroni-corrected post hoc comparisons indicated that the ankle plantar-flexion angle increased significantly from baseline to post-training (*p* = 0.027; d = 1.44).

The leg extension angle did not differ between time-points on the feedback side (F = 1.40, *p* = 0.283; η² = 0.19) or the non-feedback side (χ² = 3.43, *p* = 0.180; W = 0.24).

### Statistical parametric mapping analysis of gait kinematics

**Fig. 3:**
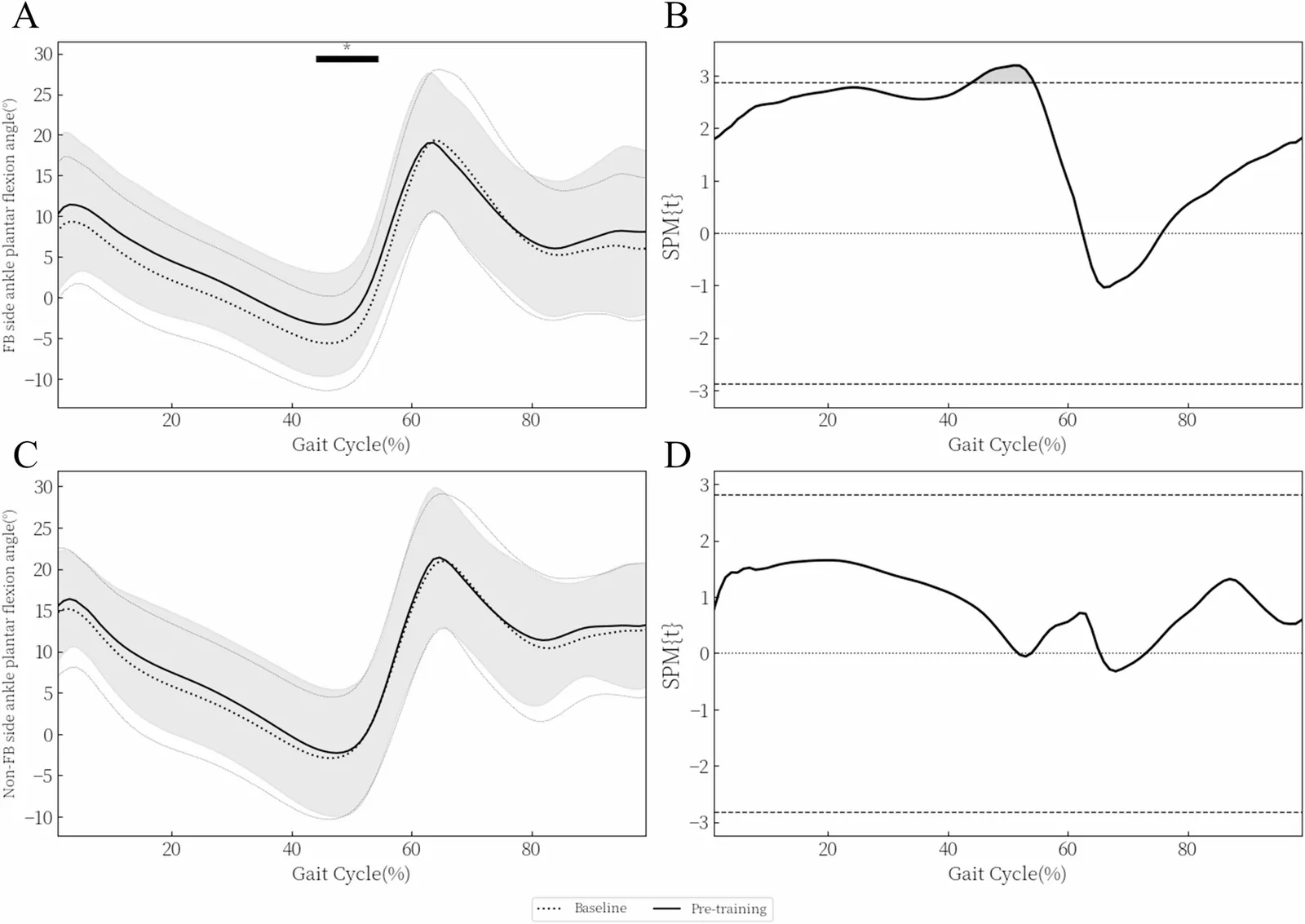
SPM1d analysis of ankle plantarflexion angle across the gait cycle during the control period. The left and right columns show the results of joint angle of time-series data on the feedback side (**A**) and the non-feedback side (**C**), and statistics of time-series data on the feedback side (**B**) and the non-feedback side (**D**), respectively. In the left columns, dot lines and bold solid lines represent mean values of at the baseline and pre-training measurements, respectively. Shade areas and solid gray lines also represent standard deviation. Black bar and asterisk in the left columns and shade area in the right columns indicate the lines represent significant differences during the control period, respectively. SPM1d, One-dimensional Statistical Parametric Mapping; FB side, Feedback side; Non-FB side, Non-feedback side

**Fig. 4:**
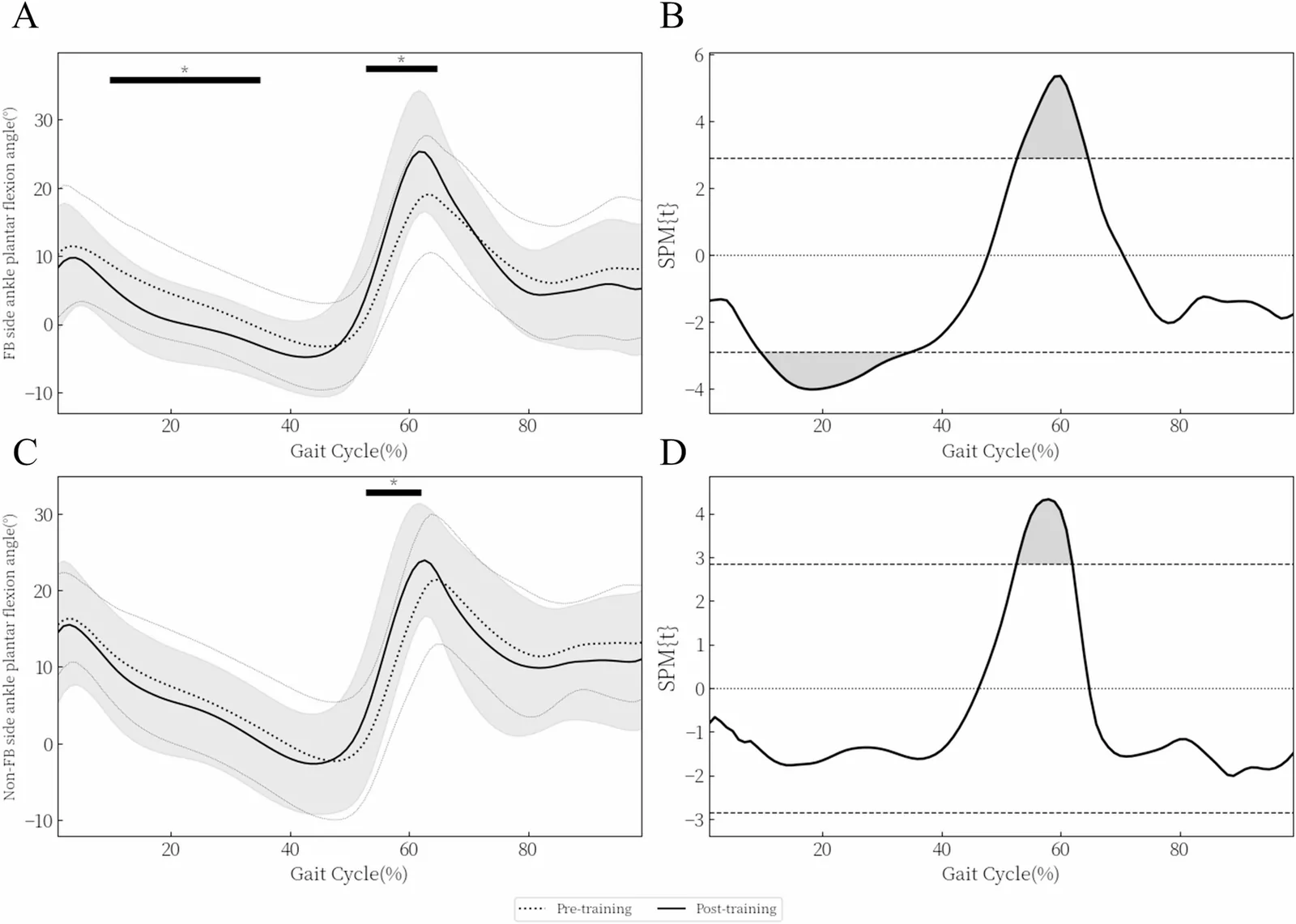
SPM1d analysis of ankle plantarflexion angle across the gait cycle during the gait training period. The left and right columns show the results of joint angle of time-series data on the feedback side (**A**) and the non-feedback side (**C**), and statistics of time-series data on the feedback side (**B**) and the non-feedback side (**D**), respectively. In the left columns, dot lines and bold solid lines represent mean values of at the pre- and post-training measurements, respectively. Shade areas and solid gray lines also represent standard deviation. Black bars and asterisks in the left columns and shade areas in the right columns indicate the lines represent significant differences during the gait training period, respectively. SPM1d, One-dimensional Statistical Parametric Mapping; FB side, Feedback side; Non-FB side, Non-feedback side

**Fig. 5:**
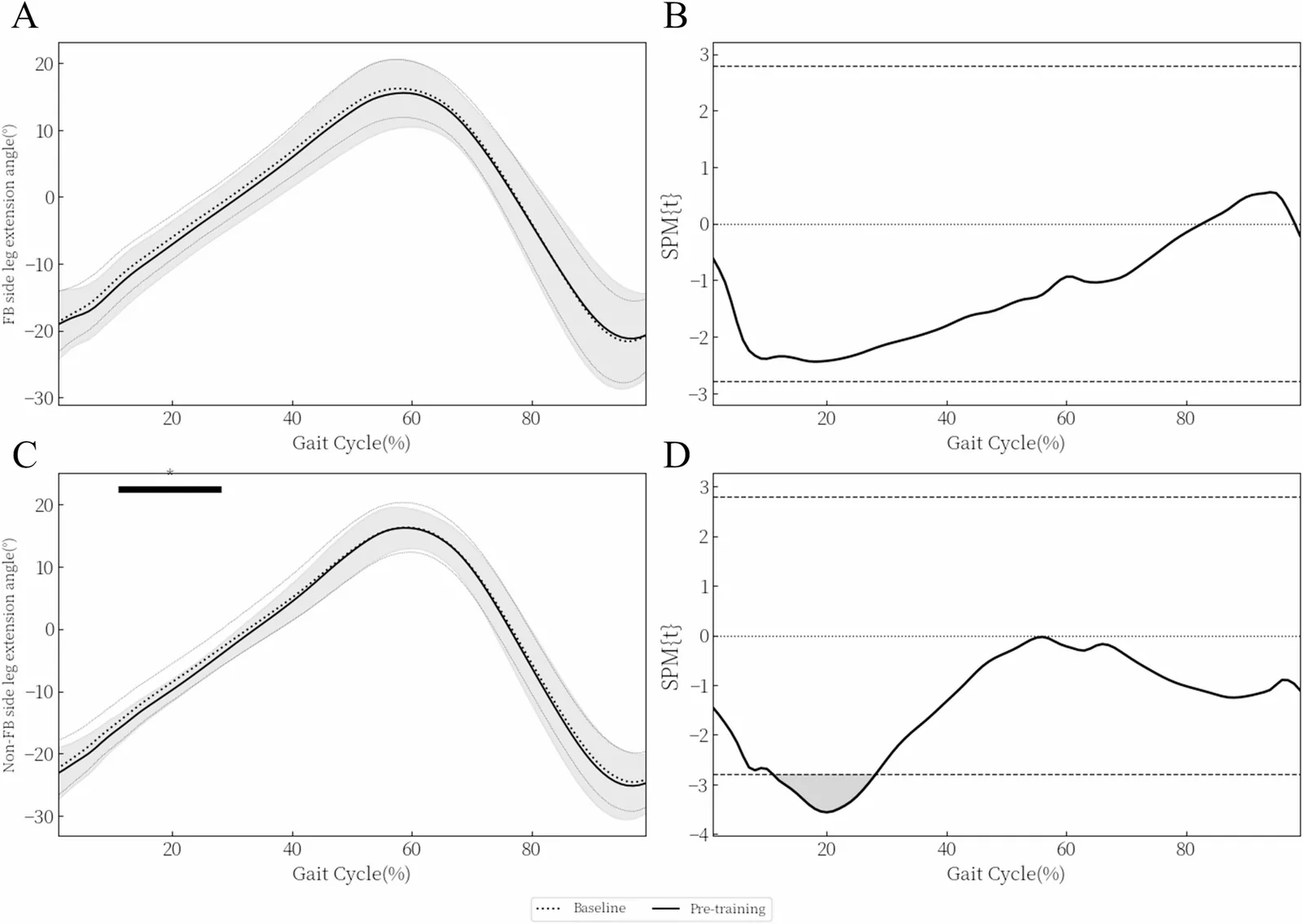
SPM1d analysis of leg extension angle across the gait cycle during the control period. The left and right columns show the results of joint angle of time-series data on the feedback side (**A**) and the non-feedback side (**C**), and statistics of time-series data on the feedback side (**B**) and the non-feedback side (**D**), respectively. In the left columns, dot lines and bold solid lines represent mean values of at the baseline and pre-training measurements, respectively. Shade areas and solid gray lines also represent standard deviation. Black bar and asterisk in the left columns and shade area in the right columns indicate the lines represent significant differences during the control period, respectively. SPM1d, One-dimensional Statistical Parametric Mapping; FB side, Feedback side; Non-FB side, Non-feedback side

**Fig. 6:**
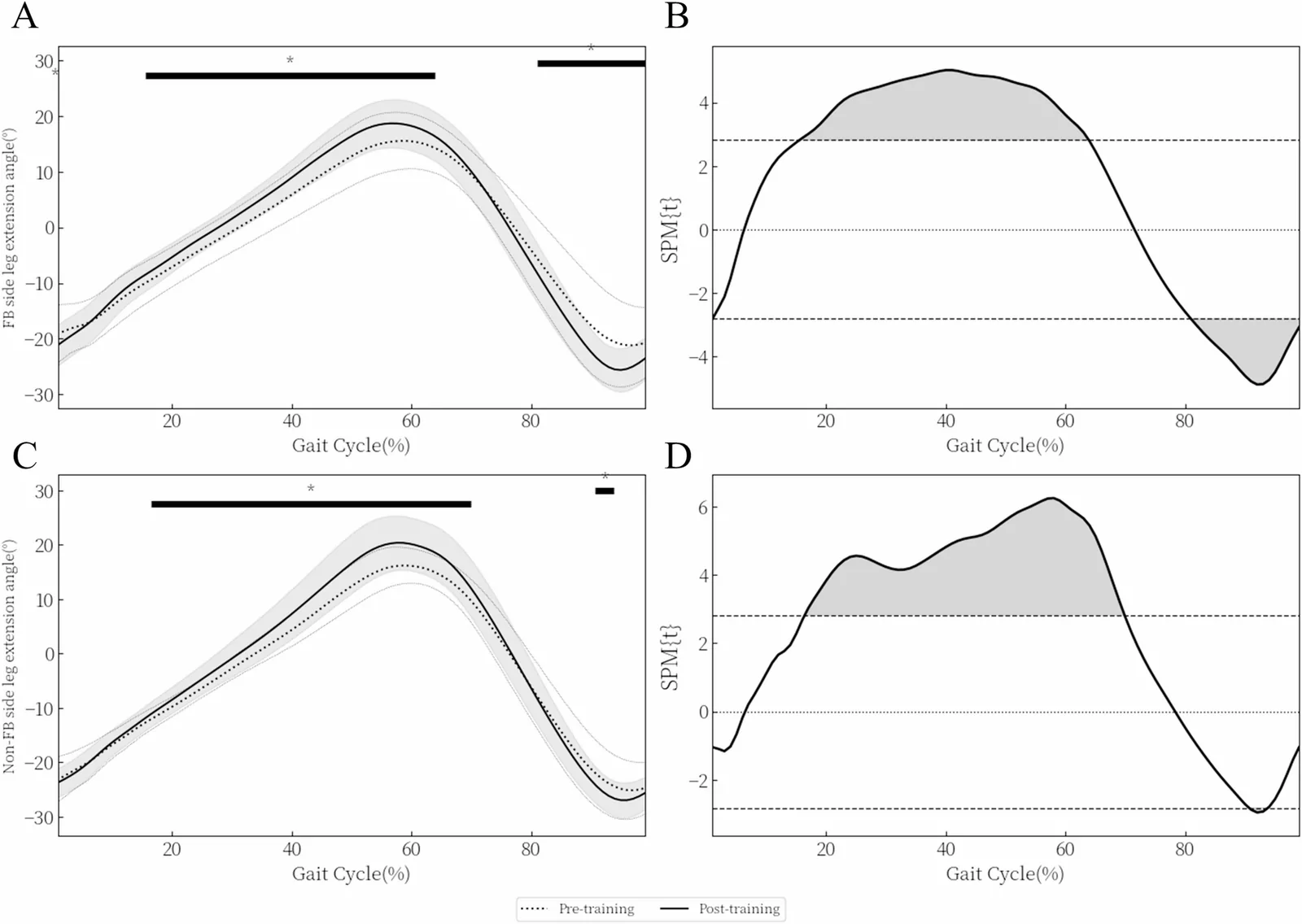
SPM1D analysis of leg extension angle across the gait cycle during the gait training period. The left and right columns show the results of joint angle of time-series data on the feedback side (**A**) and the non-feedback side (**C**), and statistics of time-series data on the feedback side (**B**) and the non-feedback side (**D**), respectively. In the left columns, dot lines and bold solid lines represent mean values of at the pre- and post-training measurements, respectively. Shade areas and solid gray lines also represent standard deviation. Black bars and asterisks in the left columns and shade areas in the right columns indicate the lines represent significant differences during the gait training period, respectively. SPM1d, One-dimensional Statistical Parametric Mapping; FB side, Feedback side; Non-FB side, Non-feedback side

The results of the SPM1d t-tests are presented in Figs. 3, 4, 5 and 6.

For the ankle plantar-flexion angle during the non-intervention period, a significant cluster was observed on the feedback side during 43%–54% of the gait cycle (Fig. 3A and B), whereas no significant clusters were found on the non-feedback side (Fig. 3C and D).

For the ankle plantar-flexion angle during the feedback period, two significant clusters were observed on the feedback side at 9%–34% and 52%–64% of the gait cycle (Fig. 4A and B), while one significant cluster was observed on the non-feedback side at 52%–61% of the gait cycle (Fig. 4C and D).

For the leg extension angle during the non-intervention period, a significant cluster was observed on the non-feedback side during 10%–28% of the gait cycle (Fig. 5C and D), whereas no significant clusters were found on the feedback side (Fig. 5A and B).

For the leg extension angle during the feedback period, two significant clusters were observed on the feedback side during 15%–63% and 80%–99% of the gait cycle (Fig. 6A and B), while two significant clusters were observed on the non-feedback side during 16%–69% and 90%–93% of the gait cycle (Fig. 6C and D).

### Relationships between gait parameters and physical function

Changes in leg extension angle during the feedback period were positively correlated with SPPB scores (*r* = 0.80, *p* = 0.032) and negatively correlated with the FTSST time (rs = -0.85; *p* = 0.015). In addition, changes in the ankle plantar-flexion angle during the feedback period were positively correlated with baseline cadence (*r* = 0.88; *p* = 0.009).

### Changes in self-efficacy

mGES did not differ across baseline, pre-training, and post-training assessments (F = 1.82, *p* = 0.204; η² = 0.23).

## Discussion

This pilot study investigated the short-term effects of real-time feedback gait training using IMUs on gait parameters in older inpatients using a within-subject design that incorporated both control and intervention periods. Gait speed increased during the feedback period, with large effect sizes. In addition, SPM1d analysis revealed changes in time-series gait kinematics, including ankle plantar-flexion angle and leg extension angle, were more pronounced than peak values and were particularly noticeable in the late stance phase. Furthermore, changes in the leg extension angle during the intervention period were correlated with physical function, as assessed by the SPPB and FTSST. These preliminary findings suggest that feedback-based gait training has the potential to alter both spatiotemporal and kinematic gait parameters, providing initial insights into the feasibility and potential role of this approach for older inpatients in clinical settings.

### Changes in spatiotemporal gait parameters

Gait speed and stride length increased after feedback gait training with large effect sizes. Cadence also differed significantly between time-points, with a large effect size, whereas the post hoc tests did not show differences. Previous studies did not show a significant increase in gait speed immediately following IMU-based feedback gait training in clinical settings [17, 18]. The improvement in gait speed observed in this study was approximately 0.14 m/s, which is comparable to the recommended criterion for a substantial meaningful change (0.10 m/s) and exceeds the threshold for a small meaningful change (0.05 m/s) in older adults [30]. Although the intervention period was limited to one week, the observed change suggests that the IMU-based biofeedback gait training may provide a clinically meaningful improvement in gait speed. Notably, this improvement was observed after five days of feedback training, suggesting that repeated practice over multiple sessions may be more effective than a single session for promoting motor learning consolidation. Gait speed can be increased by modifying cadence, stride length, or both [31]. In addition, increased stride length has been associated with improved gait symmetry [32] and reduced fall risk [14]. Considering the small sample size, these findings should be interpreted with caution. Nevertheless, the magnitude and direction of the changes may indicate a potential effect of feedback-based gait training on spatiotemporal gait parameters in older inpatients. These observed changes could provide a baseline insight into how modified gait patterns might potentially relate to increased gait speed.

### Changes in gait kinematic parameters

The mean of ankle plantar-flexion angle on the feedback side differed significantly between time points, but post hoc tests did not show statistically significant differences. Likewise, the mean of leg extension angle did not differ between time points. These findings indicate that the discrete kinematic outcomes showed only limited evidence of change. In contrast, SPM1d analysis revealed changes in time-series gait kinematics, such as ankle plantarflexion and leg extension angles on the feedback side, particularly noticeable at the late stance phase. Because discrete variables summarize only selected features (e.g., peak values) of the gait cycle, they may not capture localized temporal changes within the movement pattern. Therefore, the SPM1d findings should be interpreted as complementary to, rather than stronger than, the discrete analyses. These preliminary findings suggest that IMU-based feedback gait training may influence the timing of gait kinematics during specific phases of the gait cycle, rather than altering discrete kinematic measures such as peak values. These phase-specific changes in gait kinematics may contribute to increasing gait speed, although this interpretation requires confirmation in larger studies.

SPM1d analysis revealed significant clusters during the late stance phase of the gait cycle, indicating that alterations in joint kinematics occurred primarily during the push-off phase. In contrast, changes during the control period were minimal compared to those during the intervention period. The late stance phase, which encompasses the terminal stance and pre-swing, is a critical period for generating the propulsive forces that drive forward progression [33]. Older adults exhibit changes in gait patterns with aging, including a distal-to-proximal redistribution of joint work, with reduced ankle push-off and increased reliance on proximal muscles such as hip extensors [34]. Changes in time-series gait kinematics during the feedback period, ankle plantarflexion angle showed two significant clusters were observed on the feedback side during 9%–34% and 52%–64% of the gait cycle, and the leg extension angle also showed two significant clusters were observed on the feedback side during 15%–63% and 80%–99% of the gait cycle. These findings showed that both ankle plantar-flexion angle and leg extension angle increased during the late stance phase. Therefore, this pilot study provides preliminary evidence that feedback gait training may expand proximal and distal joint motions during gait, which could potentially contribute to increased gait speed by facilitating forward propulsion in older inpatients.

Interestingly, this pilot study also demonstrated that potential changes in time-series gait kinematics on both the feedback and non-feedback sides were more pronounced than peak values and were particularly noticeable in the late stance phase within the training period. These preliminary findings might provide an early indication of motor learning processes extending across the gait cycle during feedback-based gait training. While requiring validation in larger samples, these preliminary results tentatively suggest that the feedback-based gait training could offer potential insights into modulating gait kinematics in a phase-specific manner, particularly within the late stance phase required for forward propulsion. In this study, gait training was conducted using auditory feedback to promote an increase in joint angles during walking. There are various types of feedback [35], and our feedback system consists of extrinsic feedback, in which the knowledge of the result is provided by auditory stimulation. Auditory stimulation is reported to be more effective than visual stimulation for dynamic postural control [36], and our system uses auditory stimulation, can be implemented with a PC and IMUs, making it easy to use in a clinical setting. In this gait training, participants were also instructed to consciously modify their gait patterns even on the non-feedback side. This instruction may have contributed to the observed increases in the leg extension angle and ankle plantar-flexion angle on both the feedback and non-feedback sides after training. In contrast to our results, previous studies reported an immediate increase in the lower limb extension angle only on the feedback side, yielding results that differ from those of this study [37]. Interlimb transfer is known to be proportional to the amount of learning achieved in the trained limb [38], and reduced feedback frequencies have been shown to affect motor learning outcomes [39]. Combined with these mechanisms, our preliminary results suggest that even within the short one-week intervention period, the intervention may have facilitated the early stages of motor learning, a phase characterized by the reorganization of the task-specific motor patterns inherent to walking, rather than their simple amplification. Although this pilot study is limited by a small sample size of seven participants and a short, one-week intervention that was insufficient to confirm long-term retention, these findings offer promising preliminary evidence regarding the feasibility and potential efficacy of task-specific gait training designed to promote motor learning of walking for this population.

### Correlation between physical function and gait parameters

The leg extension angle during the feedback period was positively correlated with SPPB scores and negatively correlated with FTSST time, although these correlations must be interpreted with caution due to the small sample size, these results nevertheless offer a promising insight suggesting that appropriate gait training indicators may vary depending on the inpatient’s baseline physical function. Additionally, changes in ankle plantar-flexion angle during the feedback period were positively correlated with baseline cadence. This suggests that older inpatients with higher baseline cadence may be more likely to increase their ankle plantar-flexion angle with gait training. To increase gait speed, individuals can either increase their cadence or stride length, or use a combination of both strategies [31, 40]. Among older adults at risk of mobility disability, those who primarily increased stride length to increase gait speed demonstrated better physical function (shorter FTSST time) than those who primarily increased cadence [40]. Another study reported that when physical function declines with age, cadence tends to increase when walking at the same speed as younger individuals [41]. Despite the sample size constraints, this pilot study provides preliminary insights into the potential importance of considering individual physical function and kinematic characteristics when selecting gait training targets. Whether tailoring gait training targets according to these characteristics improves the efficacy of feedback-based gait training in clinical settings should be confirmed in future studies.

### Changes in self-efficacy

No significant differences in mGES were found between time-points. This suggests that, despite improvements in gait speed and kinematics following feedback gait training, participants’ self-efficacy regarding walking did not change. This may be because the intervention period was short, and changes in self-efficacy may require longer intervention periods. Additionally, because the mGES encompasses various advanced walking activities such as stair climbing and walking on uneven surfaces [24], flat-ground gait training within a short-term may be insufficient for immediate score improvements. However, over a longer period, sustained improvements in gait speed and stability might gradually enhance self-efficacy for more complex walking tasks. Since this pilot study did not include a long-term follow-up assessment, further longitudinal research with extended training periods is needed to investigate whether chronic gait adaptations could potentially influence self-efficacy in diverse, real-world environments.

### Study limitations and future directions

This pilot study had some limitations. First, although SPM1d provided detailed temporal information across the gait cycle, the sample size was small (*n* = 7) and included participants with different underlying conditions and functional levels, which limited statistical power and generalizability. Although the within-subject design with a control period was employed to mitigate inter-individual variability and optimize the detection of effects within a small sample size, this exploratory study is still limited by the lack of a parallel control group. Without a parallel control group, the contribution of the intervention to the observed changes cannot be determined with certainty. Second, participants in this study had diverse diagnoses and levels of physical function, yet the analyses treated them as a single group. Heterogeneity in underlying conditions among participants may have influenced their gait characteristics and responsiveness to the intervention. Furthermore, because this pilot study used two different joint angles as indicators of gait training, the effectiveness of the gait training may have varied between participants. Therefore, future studies should focus on utilizing a single unified joint angle as an indicator to independently verify the specific efficacy of each feedback-based gait training, while also identifying the clinical characteristics associated with responders and non-responders to this gait training. Third, regarding the feedback modality, the auditory stimulation used in this study does not provide the exact numerical value of the joint angle required for correction. Therefore, further investigation is required to establish appropriate instructional and feedback protocols for gait training. Future studies should encompass comparative evaluations with other modalities, such as vibrotactile stimulation, while systematically examining the impact of different feedback frequencies and training doses. Finally, the short, one-week duration of the intervention limits conclusions regarding long-term effects. Furthermore, because physical functions such as the SPPB and FTSST were not assessed at the endpoint, future studies must follow up and track these outcomes to evaluate whether this gait training leads to meaningful improvements in physical functions. These limitations highlight the need for larger, well-controlled studies to confirm these preliminary findings.

Future studies with larger sample sizes are warranted to confirm the present findings and improve generalizability. In addition, studies with more homogeneous participant characteristics may help clarify the effects of feedback-based gait training on specific populations. The use of randomized controlled designs would further strengthen causal inferences regarding the effectiveness of the intervention. Moreover, longer intervention and follow-up periods are required to determine the sustainability of the observed changes. Finally, future studies incorporating both discrete parameters and waveform-level analyses, such as SPM1d, may provide a more comprehensive understanding of gait adaptation.

## Conclusion

Feedback gait training using IMUs may increase gait speed by altering gait kinematics in older inpatients in clinical settings. In addition, changes in time-series gait kinematics, such as ankle plantarflexion and leg extension angles on the feedback side, particularly noticeable at the late stance phase. Given the small sample size and short-term exploratory nature of this pilot study, these findings should be interpreted with caution. Nevertheless, these results provide preliminary insights into the potential role of feedback-based gait training for older inpatients in clinical settings.

## Data Availability

The datasets used and/or analyzed during the current study are available from the corresponding author upon reasonable request.

## Abbreviations

IMU: Inertial Measurement Unit
SPPB: Short Physical Performance Battery
FTSST: Five-Time Sit-to-Stand Test
mGES: modified Gait Efficacy Scale
SPM1d: One-dimensional Statistical Parametric Mapping
M: Male
F: Female
LEA: Leg Extension Angle
ANK: Ankle plantarflexion angle

## References

[1] van Abellan G, Rolland Y, Andrieu S, Bauer J, Beauchet O, Bonnefoy M, Cesari M, Donini LM, Gillette Guyonnet S, Inzitari M, et al. Gait speed at usual pace as a predictor of adverse outcomes in community-dwelling older people an International Academy on Nutrition and Aging (IANA) Task Force. J Nutr Health Aging. 2009;13(10):881–9. 10.1007/s12603-009-0246-z. PMC12878092 19924348

[2] Jansen CP, Klenk J, Nerz C, Todd C, Labudek S, Kramer-Gmeiner F, Becker C, Schwenk M. Association between everyday walking activity, objective and perceived risk of falling in older adults. Age Ageing. 2021;50(5):1586–92. 10.1093/ageing/afab037. PMC8437067 33710267

[3] Gordt K, Gerhardy T, Najafi B, Schwenk M. Effects of Wearable Sensor-Based Balance and Gait Training on Balance, Gait, and Functional Performance in Healthy and Patient Populations: A Systematic Review and Meta-Analysis of Randomized Controlled Trials. Gerontology. 2018;64(1):74–89. 10.1159/000481454. 29130977

[4] Beatty J, Legewie H. Biofeedback and behavior: Introduction to the Proceedings. In: *Biofeedback and behavior.* Springer; 1977: 1–5.

[5] Browne MG, Franz JR. Ankle power biofeedback attenuates the distal-to-proximal redistribution in older adults. Gait Posture. 2019;71:44–9. 10.1016/j.gaitpost.2019.04.011. PMC7897464 31005854

[6] Genthe K, Schenck C, Eicholtz S, Wolf S, Kesar T. Effects of Real-time Gait Biofeedback on Paretic Propulsion and Gait Biomechanics in Individuals Post-Stroke. Arch Phys Med Rehabil. 2017;98(10). 10.1016/j.apmr.2017.08.020. PMC5901660 29457532

[7] Santucci V, Alam Z, Liu J, Spencer J, Faust A, Cobb A, Konantz J, Eicholtz S, Wolf S, Kesar TM. Immediate improvements in post-stroke gait biomechanics are induced with both real-time limb position and propulsive force biofeedback. J Neuroeng Rehabil. 2023;20(1):37. 10.1186/s12984-023-01154-3. PMC10064559 37004111

[8] Vickery-Howe DM, Bonanno DR, Dascombe BJ, Drain JR, Clarke AC, Hoolihan B, Willy RW, Middleton KJ. Physiological, perceptual, and biomechanical differences between treadmill and overground walking in healthy adults: A systematic review and meta-analysis. J Sports Sci. 2023;41(23):2088–120. 10.1080/02640414.2024.2312481. 38350022

[9] Malatesta D, Canepa M, Menendez Fernandez A. The effect of treadmill and overground walking on preferred walking speed and gait kinematics in healthy, physically active older adults. Eur J Appl Physiol. 2017;117(9):1833–43. 10.1007/s00421-017-3672-3 28687953

[10] Hinton EH, Buffum R, Stergiou N, Kingston D, Bierner S, Knarr BA. A portable visual biofeedback device can accurately measure and improve hip extension angle in individuals post-stroke. Clin Biomech (Bristol Avon). 2023;105:105967. 10.1016/j.clinbiomech.2023.105967. PMC10198940 37087881

[11] Araki S, Matsuura H, Miyazaki T, Matsuzawa Y, Nakai Y, Kawada M, Takeshita Y, Takamura M, Kiyama R. Longitudinal changes in vertical stride regularity, hip flexion, and knee flexion contribute to the alteration in gait speed during hospitalization for stroke. Hum Mov Sci. 2024;95:103227. 10.1016/j.humov.2024.103227. 38723306

[12] Miyazaki T, Kiyama R, Nakai Y, Kawada M, Takeshita Y, Araki S, Hayashi H, Higashi N, Makizako H. The Relationship between Leg Extension Angle at Late Stance and Knee Flexion Angle at Swing Phase during Gait in Community-Dwelling Older Adults. Int J Environ Res Public Health. 2021;18(22). 10.3390/ijerph182211925. PMC8625228 34831678

[13] Miyazaki T, Kiyama R, Nakai Y, Kawada M, Takeshita Y, Araki S, Makizako H. Relationships between Gait Regularity and Cognitive Function, including Cognitive Domains and Mild Cognitive Impairment, in Community-Dwelling Older People. Healthc (Basel). 2021;9(11). 10.3390/healthcare9111571. PMC8620724 34828617

[14] Mao Q, Zhang J, Yu L, Zhao Y, Luximon Y, Wang H. Effectiveness of sensor-based interventions in improving gait and balance performance in older adults: systematic review and meta-analysis of randomized controlled trials. J Neuroeng Rehabil. 2024;21(1):85. 10.1186/s12984-024-01375-0. PMC11131332 38807117

[15] Miyazaki T, Takeshita Y, Shimose D, Kakimoto S, Araki S, Matsuzawa Y, Nakashima S, Nakai Y, Kawada M, Kiyama R. Immediate Effects of Real-Time Feedback During Overground Gait Performed Using Inertial Measurement Units on Gait Parameters in Healthy Young Participants: A Cross-Sectional Study. Motor Control. 2023;27(4):844–59. 10.1123/mc.2022-0122. 37487588

[16] Araki S, Miyazaki T, Shibasaki J, Okumura K, Ishii A, Shimose D, Takeshita Y, Takamura M, Kiyama R. Examination of effect and responder to real-time auditory feedback during overground gait for stroke: a randomized cross-over study. Sci Rep. 2025;15(1):8519. 10.1038/s41598-025-93262-4. PMC11904209 40074803

[17] Hinton EH, Buffum R, Kingston D, Stergiou N, Kesar T, Bierner S, Knarr BA. Real-Time Visual Kinematic Feedback During Overground Walking Improves Gait Biomechanics in Individuals Post-Stroke. Ann Biomed Eng. 2024;52(2):355–63. 10.1007/s10439-023-03381-0. PMC11010657 37870663

[18] Miyazaki T, Kiyama R, Takeshita Y, Shimose D, Araki S, Matsuura H, Uto Y, Nakashima S, Nakai Y, Kawada M. Inertial measurement unit-based real-time feedback gait immediately changes gait parameters in older inpatients: a pilot study. Front Physiol. 2024;15:1384313. 10.3389/fphys.2024.1384313. PMC11333335 39165280

[19] Browne MG, Franz JR. More push from your push-off: Joint-level modifications to modulate propulsive forces in old age. PLoS ONE. 2018;13(8):e0201407. 10.1371/journal.pone.0201407. PMC6082565 30089143

[20] Hsiao H, Awad LN, Palmer JA, Higginson JS, Binder-Macleod SA. Contribution of Paretic and Nonparetic Limb Peak Propulsive Forces to Changes in Walking Speed in Individuals Poststroke. Neurorehabil Neural Repair. 2016;30(8):743–52. 10.1177/1545968315624780. PMC4930429 26721869

[21] Guralnik JM, Ferrucci L, Pieper CF, Leveille SG, Markides KS, Ostir GV, Studenski S, Berkman LF, Wallace RB. Lower extremity function and subsequent disability: consistency across studies, predictive models, and value of gait speed alone compared with the short physical performance battery. J Gerontol Biol Sci Med Sci. 2000;55(4):M221–231. 10.1093/gerona/55.4.m221. PMC12149745 10811152

[22] Albalwi AA, Alharbi AA. Optimal procedure and characteristics in using five times sit to stand test among older adults: A systematic review. Med (Baltim). 2023;102(26):e34160. 10.1097/MD.0000000000034160. PMC10313281 37390277

[23] Makizako H, Shimada H, Park H, Doi T, Yoshida D, Uemura K, Tsutsumimoto K, Suzuki T. Evaluation of multidimensional neurocognitive function using a tablet personal computer: test-retest reliability and validity in community-dwelling older adults. Geriatr Gerontol Int. 2013;13(4):860–6. 10.1111/ggi.12014. 23230988

[24] Newell AM, VanSwearingen JM, Hile E, Brach JS. The modified Gait Efficacy Scale: establishing the psychometric properties in older adults. Phys Ther. 2012;92(2):318–28. 10.2522/ptj.20110053. PMC3269773 22074940

[25] Revi DA, Alvarez AM, Walsh CJ, De Rossi SMM, Awad LN. Indirect measurement of anterior-posterior ground reaction forces using a minimal set of wearable inertial sensors: from healthy to hemiparetic walking. J Neuroeng Rehabil. 2020;17(1):82. 10.1186/s12984-020-00700-7. PMC7322880 32600348

[26] Miyazaki T, Kawada M, Nakai Y, Kiyama R, Yone K. Validity of Measurement for Trailing Limb Angle and Propulsion Force during Gait Using a Magnetic Inertial Measurement Unit. Biomed Res Int. 2019;2019:8123467. 10.1155/2019/8123467. PMC6942796 31930138

[27] Cohen J. Statistical power analysis for the behavioral sciences. routledge; 2013.

[28] Pataky TC. rft1d: Smooth One-Dimensional Random Field Upcrossing Probabilities inPython. J Stat Softw. 2016;71(7). 10.18637/jss.v071.i07.

[29] Pataky TC, Robinson MA, Vanrenterghem J. Vector field statistical analysis of kinematic and force trajectories. J Biomech. 2013;46(14):2394–401. 10.1016/j.jbiomech.2013.07.031. 23948374

[30] Perera S, Mody SH, Woodman RC, Studenski SA. Meaningful change and responsiveness in common physical performance measures in older adults. J Am Geriatr Soc. 2006;54(5):743–9. 10.1111/j.1532-5415.2006.00701.x. 16696738

[31] Howard C, Wallace C, Stokic DS. Stride length-cadence relationship is disrupted in below-knee prosthesis users. Gait Posture. 2013;38(4):883–7. 10.1016/j.gaitpost.2013.04.008. 23684100

[32] Balasubramanian CK, Bowden MG, Neptune RR, Kautz SA. Relationship between step length asymmetry and walking performance in subjects with chronic hemiparesis. Arch Phys Med Rehabil. 2007;88(1):43–9. 10.1016/j.apmr.2006.10.004. 17207674

[33] Zelik KE, Adamczyk PG. A unified perspective on ankle push-off in human walking. J Exp Biol. 2016;219(Pt 23):3676–83. 10.1242/jeb.140376. PMC5201006 27903626

[34] DeVita P, Hortobagyi T. Age causes a redistribution of joint torques and powers during gait. J Appl Physiol (1985). 2000;88(5):1804–11. 10.1152/jappl.2000.88.5.1804. 10797145

[35] Sigrist R, Rauter G, Riener R, Wolf P. Augmented visual, auditory, haptic, and multimodal feedback in motor learning: a review. Psychon Bull Rev. 2013;20(1):21–53. 10.3758/s13423-012-0333-8. 23132605

[36] Hasegawa N, Takeda K, Mancini M, King LA, Horak FB, Asaka T. Differential effects of visual versus auditory biofeedback training for voluntary postural sway. PLoS ONE. 2020;15(12):e0244583. 10.1371/journal.pone.0244583. PMC7769480 33370408

[37] Schenck C, Kesar TM. Effects of unilateral real-time biofeedback on propulsive forces during gait. J Neuroeng Rehabil. 2017;14(1):52. 10.1186/s12984-017-0252-z. PMC5460355 28583196

[38] Krishnan C. Learning and interlimb transfer of new gait patterns are facilitated by distributed practice across days. Gait Posture. 2019;70:84–9. 10.1016/j.gaitpost.2019.02.019. PMC6474794 30831544

[39] Marco-Ahullo A, Villarrasa-Sapina I, Romero-Martinez J, Monfort-Torres G, Toca-Herrera JL, Garcia-Masso X. Effect of Reduced Feedback Frequencies on Motor Learning in a Postural Control Task in Young Adults. Sens (Basel). 2024;24(5). 10.3390/s24051404. PMC10933749 38474940

[40] Baudendistel ST, Schmitt AC, Stone AE, Raffegeau TE, Roper JA, Hass CJ. Faster or longer steps: Maintaining fast walking in older adults at risk for mobility disability. Gait Posture. 2021;89:86–91. 10.1016/j.gaitpost.2021.07.002. PMC9277656 34256264

[41] Larish DD, Martin PE, Mungiole M. Characteristic patterns of gait in the healthy old. Ann N Y Acad Sci. 1988;515:18–32. 10.1111/j.1749-6632.1988.tb32960.x. 3364884

